# About Correct

**Published:** 1890-02

**Authors:** 


### ABOUT CORRECT.

It seems as if our remarks regarding the inconsiderate treatment of the published statements of Dr. Brown-Séquard and our prediction of a change of front in the near future upon the part of the medical press is being realized, for in an editorial in a recent number of the *American Practitioner and News* we note the following:

"That there is a germ of truth in the apparently absurd claims of Brown-Séquard would seem to be attested by the following item, which is just now going the rounds in the medical press: '*Spermine* is an alkaloid found in the *testicular juice*, in the gray matter of the brain, in eggs, oysters, lampreys, fish ova and milt, also in the products of all atonic mucous membranes. It also appears in the sputa of senile and acute bronchitis, in the expectoration of phthisis, and of emphysema with catarrh, and in the spleen and circulation of anæmias and of leucocythæmias. The *hydrochlorate of spermine*, in a dose of one-fortieth of a grain, injected subcutaneously in a dog of thirteen pounds produced marked mental and physical activity, and powerful and prolonged stimulation of the genital system.'

"We don't know the age of this item, as we have not seen the original article from which it was culled, but take it to be a recent physiologico-chemical investigation proving the competency of testicular juice to supply to the devitalized a vitalizing compound, which, if the alkaloid can be isolated in a pure state, may prove to be a therapeutic agent of real stimulant or constructive force and of easy administration.

"At any rate, it begins to look as if the great Franco-American physiologist was about to turn the laugh on those who have been in too great a hurry to laugh at him."

"Truth crushed to earth will rise again," never fear; a few more such articles as the above from which we have quoted, and those who have made honest investigations with the Brown-Séquard "elixir," will come forward and publish the results of their experimentation in that direction, which have been kept back for fear of being classed with the quacks and charlatans who rushed into print in the secular press. Let us hear from others.

